# Examining the Efficacy and Results of a Short-Form Alzheimer's Survey With College Students

**DOI:** 10.1093/geroni/igab046.1882

**Published:** 2021-12-17

**Authors:** Debra Valencia-Laver, Brooke Buchanan, Chelsea McPheron, Anna Rogers, Alex DeTurck, Jasmine Shapiro, Gary Laver

**Affiliations:** 1 California Polytechnic State University, San Luis Obispo, Cal Poly/San Luis Obispo, California, United States; 2 California Polytechnic State University, San Luis Obispo, Cal Poly, San Luis Obispo, California, United States

## Abstract

College students are important stakeholders in addressing the significant costs of Alzheimer’s disease in their future roles as caretakers, health care consumers, taxpayers, and as individuals in the workforce whose careers may interact with and impact those with Alzheimer’s and their caregivers. To assess their knowledge of Alzheimer’s, a 10-item True/False on-line quiz was presented to 912 students in Introductory Psychology classes. Participants were 61% white, 13% Asian/Asian American, and 10% Latinx, with 14% reporting other racial and ethnic groups, including that of mixed heritage; 59% of the sample self-reported as female. The quiz was counterbalanced such that items appearing in one format (e.g., True) appeared in the other format (e.g., False) across the two forms of the quiz. A significant difference was found for percent correct in Form A (61.4%) versus Form B (59.3%). In order to prompt participants to consider the ways the disease may impact their own lives, additional questions examined students’ own experience with Alzheimer’s, their interest and willingness to take action towards supporting Alzheimer’s research, and their perceptions about how Alzheimer’s would impact their lives personally, financially, and in their career pursuits. The research extends the findings of earlier research on student knowledge of Alzheimer’s (e.g., Bailey, 2000; Eshbaugh, 2014) by allowing the results to be broken down by gender, race/ethnicity, and student major. It also expands upon those findings by identifying how college students project the societal effects and costs of Alzheimer’s to their own lives and livelihoods.

